# To assess the effectiveness of Systane COMPLETE in improving meibomian gland score and in reducing symptoms of ocular dryness

**DOI:** 10.3389/fopht.2025.1577836

**Published:** 2025-11-06

**Authors:** Namrata Sharma, Aafreen Bari, Anu Malik, Prafulla Kumar Maharana, Chetan Shakkarwal, Shivam Sharma, Aishwarya Dasgupta

**Affiliations:** Dr. R. P. Centre for Ophthalmic Sciences, All India Institute of Medical Sciences, New Delhi, India

**Keywords:** dry eye disease (DED), Systane COMPLETE, tear break up time (TBUT), Schirmer, Meibo Score

## Abstract

**Purpose:**

To assess the effectiveness of Systane COMPLETE in reducing symptoms of ocular dryness and improving Meibomian Gland (MG) Score in subjects with mild to moderate dry eye disease (DED).

**Methods:**

A prospective, interventional, single-center, single-arm study was conducted including cases of mild to moderate DED. Participants were prescribed a topical lipid-based combination of propylene glycol (PG) and hydroxypropyl guar (HPG) (Systane^®^ COMPLETE), administered four times daily for 30 days. Dry eye assessment parameters, Meibo score, and Ocular Surface Disease Index (OSDI) questionnaire responses were obtained before and after treatment.

**Results:**

A total of 105 cases with mild to moderate DED were included in the study. Statistically significant improvements were observed in tear breakup time (TBUT) (p<0.0001), Schirmer’s test (p<0.0001), lipid layer thickness (LLT) (p<0.0001), tear meniscus height (TMH) (p=0.0002), non-invasive breakup time (NIBUT) (p<0.0001), OSDI (p<0.0001), upper lid meibomian gland score (p=0.02), corneal staining score (p<0.0001). The change in Meibo score was not statistically significant (p=0.19).

**Conclusion:**

A lipid-based nanoemulsion of PG–HPG helps in improving the objective parameters and symptoms of DED. Additionally, it may support improved meibomian gland function.

## Introduction

Dry eye disease (DED) is one of the most common lifestyle disorders affecting the eye. The underlying etiology may be variable and dependent on many factors. The dry eye workshop (DEWS) II associates it with loss of homeostasis of the tear film, tear film instability, ocular surface inflammation and neurosensory abnormalities (1).

Management of DED requires understanding its etiopathogenesis and classifying the disease as evaporative, aqueous-deficient, or mixed type, in order to guide treatment. First-line therapy often includes lubricants such as carboxymethyl cellulose (CMC), hydroxypropyl methylcellulose (HPMC), polyethylene glycol (PEG), and polypropylene glycol (PPG) (2). Other therapeutic classes include anti-inflammatory agents such as corticosteroids and immunomodulators like cyclosporine and tacrolimus. However, immunomodulators are not uniformly approved for the treatment of DED.

Newer treatment options include lifitegrast, which disrupts the inflammatory cascade by blocking lymphocyte function-associated antigen-1 (LFA-1), and varenicline, which stimulates tear production by acting on the trigeminal parasympathetic pathway (3–6).

Previously, in cases of evaporative-type and mild to moderate grades of DED, artificial tears were considered the gold standard (7). However, with the development of newer and more advanced lipid-based formulations, the ideal choice of lubricant drops for evaporative DED remains debatable. Several studies have demonstrated that lipid-based nanoemulsions exhibit superior proficiency in stabilizing the tear film compared to non-lipid emollients (8–12).

Comparative studies in the literature have assessed various lipid-based lubricant eye drops. In a randomized non-inferiority trial, Jerkins et al. evaluated the efficacy of Systane Balance^®^ versus Refresh^®^ Optive Advanced. They concluded that both groups were comparable in improving tear breakup time (TBUT) and alleviating symptoms of ocular discomfort (13).

Systane^®^ COMPLETE (Alcon Laboratories, Inc., Fort Worth, TX, USA) is a sterile emulsion containing propylene glycol (PG), hydroxypropyl guar (HPG), mineral oil, dimyristoyl phosphatidylglycerol, polyoxyl 40 stearate, sorbitan tristearate, boric acid, sorbitol, edetate disodium, POLYQUAD™ (polyquaternium-1) 0.001% preservative, and purified water. The 0.6% PG acts as a demulcent that functions as a lubricant. HPG interacts with borate ions in the tear film to form a soft, thin, cross-linked *in situ* gel matrix. This gel structure improves the retention of the lubricant on the ocular surface, thereby providing prolonged protection and hydration. Additionally, dimyristoyl phosphatidylglycerol (DMPG) is an anionic phospholipid that plays a vital role in restoring the lipid layer of the tear film. The nanoemulsion-based delivery system thereby facilitates sustained release of DMPG, improving its distribution across the ocular surface for enhanced coverage and therapeutic efficacy (14).

Hence, lipid-based eye drops effectively stabilize the lipid layer of the tear film. Their formulation includes phospholipids and smaller particle sizes, allowing them to bind to epithelial cells and form a gel-like matrix with a mesh-like network. This structure provides better ocular surface coverage while minimizing blurring.

In the initial studies, preclinical evaluation of phospholipid nanoemulsion-based lubricant eye drops was performed using *in vitro* and ex vivo corneal epithelial models. The percentage protection provided by the lipid nanoemulsion solution was compared to controls. It was observed that, compared to the control group, the cells treated with phospholipid nanoemulsion showed better protection against desiccation. The nanoemulsion group had greater moisture retention and superior cellular barrier function (15).

The effect of these drops on the Meibomian glands has not been studied previously. Therefore, a study was planned with the aim of evaluating the effect of lipid-based nanoemulsion in mild to moderate grades of evaporative dry eye disease. Meibomian gland health was graded using objective parameters (meiboscore). The improvement in lid margin anatomy, upper and lower lid gland anatomy, expression of the glands, and meibum quality was quantified and analyzed before and after usage of the lipid-based drops.

## Methodology

This was a prospective, interventional, single-center, single-arm study conducted at a tertiary eye center in India. Institutional ethics committee approval was obtained (IEC-376), and the study was registered with the Clinical Trials Registry of India (CTRI/2024/02/063319).

The study included subjects aged 18 to 55 years who presented with symptoms of dry eye disease affecting both eyes. Patients with OSDI score between 13 and 32 and a baseline MG score >20 were included. Subjects were excluded if they had severe dry eye disease (OSDI score ≥ 33), a history of ocular surgery, were using other artificial tear products, had been diagnosed with other ocular pathologies, had used contact lenses in the past 6 months, were pregnant or breastfeeding, or had a known allergy to any component of the eye drop. Both newly diagnosed and previously treated cases were included. For those already on treatment, a washout period of 2 weeks was observed. Patients were prescribed a lipid-based nanoemulsion of propylene glycol/hydroxypropyl guar (PG-HPG) Systane Complete (Alcon Laboratories, Inc. Fort Worth, TX, USA) four times daily and followed up on day 30.

Each patient underwent uncorrected distance visual acuity (UDVA) testing, Schirmer’s test, slit-lamp examination, tear breakup time (TBUT), and evaluation using an Ocular Surface Analyzer (OSA), which included measurements of lipid layer thickness (LLT), tear meniscus height (TMH), non-invasive breakup time (NIBUT), and Meibomian gland dropout of the upper and lower lids. Subjective analysis was performed using the OSDI questionnaire. The meibo score (0-12) was calculated by using five parameters: lid margin (0–3 based on irregularity, vascular engorgement and orifice obstruction); meibomian gland expressibility (0–3 based on number of glands that could be expressed), meibum score (0–3 based on consistency of meibum) and meibo score (0–3 based on area of dropouts).

### Outcome measures

The mean change in OSDI score from baseline to day 30 of follow-up was considered the primary outcome measure. The mean change in non-invasive breakup time (NIBUT) and MG score from baseline to 30-day follow-up were considered secondary outcome measures.

The results of the worse eye were included in the study. In cases where both eyes had a similar grade of dry eye disease, data from the right eye were used for analysis.

### Sample size calculation

Based on the mean (SD) OSDI score at baseline (17.7 ± 16) and the average OSDI score after using the eye drops four times daily for 1 month (12.4 ± 14.2), an effect size of 0.35099 was calculated. Assuming a study power of 90%, a significance level of 5%, and a 20% dropout rate, the required sample size was determined to be 105 patients.

### Statistical analysis

Inferential statistics (for the primary outcome measure) and descriptive statistics (for other endpoints) were used to represent demographic and clinical variables. An OSDI score change of 4.5 to 7.3 was considered meaningful for dry eye disease with mild to moderate symptoms. A p-value of less than 0.05 was considered statistically significant. Categorical variables were presented as number and percentage, and continuous variables were represented as mean and standard deviation/median(range). Change in quantitative variables were assessed by paired t- test or Wilcoxon sign rank test as appropriate. A p-value less than 0.05 was considered to be statistically significant.

## Results

A total of 105 patients with mild to moderate dry eye disease were recruited into the study. Of these, 102 patients completed the final follow-up, while three were lost to follow-up. There was a significant improvement in the mean uncorrected distance visual acuity (UDVA), from log MAR 0.22 ± 0.08 to log MAR 0.05 ± 0.09 (p=0.0005).

### Dry eye disease parameters and OSDI score

At the 1-month follow-up, tear breakup time (TBUT) improved from a mean of 2.85 ± 1.39 s to 4.18 ± 1.73 s (p<0.0001). The mean Schirmer score increased from 14.58 ± 10.47 to 18.70 ± 10.35 (p<0.0001). Lipid layer thickness (LLT) improved from 29.43 ± 2.20 to 30.0 ± 0.00 (p<0.0001). Tear meniscus height (TMH) increased from 0.22 ± 0.09 to 0.23 ± 0.07 (p=0.0002). Non-invasive breakup time (NIBUT) improved from 4.68 ± 2.35 s to 5.70 ± 2.68 s (p<0.0001). The corneal staining score decreased from 2.43 ± 2.48 to 0.76 ± 1.85 (p<0.0001).

The percentage loss of meibomian gland area in the upper lids improved at 1 month (p=0.02), while changes in the lower lids were not statistically significant (p=0.08) (**Table 1** and **Supplementary Table 1**).

**Table 1:** Dry eye disease parameters at baseline and after one month following use of Systane complete eye drops.

S.NO.	PARAMETERS	BASELINE (Mean ± S.D)	1 MONTH (Mean ± S.D)	P-value
1	TBUT	2.85 ± 1.39	4.18 ± 1.73	<0.0001
2	SCHIRMER’S TEST	14.58 ± 10.47	18.70 ± 10.35	<0.0001
3	LLT	29.43 ± 2.20	30.0 ± 0.00	<0.0001
4	TMH	0.22 ± 0.09	0.23 ± 0.07	0.0002
5	NIBUT	4.68 ± 2.35	5.70 ± 2.68	<0.0001
6	OSDI	23.22 ± 8.06	17.86 ± 8.62	<0.0001
7	MG drop out UPPER LID (LOSS %)	28.34 ± 8.20	27.62 ± 7.84	0.0242
8	MG drop out LOWER LID (LOSS %)	22.95 ± 11.27	22.97 ± 10.59	0.0838
9	CORNEAL STANING	2.43 ± 2.48	0.76 ± 1.85	<0.0001
10	MG SCORE			
	UPPER	3.30 ± 1.28	3.31 ± 1.29	0.3873
	LOWER	3.34 ± 1.22	3.19 ± 1.18	0.0021
11	LID MARGIN			
	UPPER	0.96 ± 0.53	0.92 ± 0.53	0.0332
	LOWER	1.02 ± 0.52	0.98 ± 0.52	0.0768
12	MG EXPRESSIBILITY			
	Upper	0.49 ± 0.57	0.58 ± 0.53	0.0001
	Lower	0.07 ± 0.26	0.13 ± 0.34	0.0010
13	MEIBUM SCORE	1.27 ± 0.46	1.21 ± 0.41	0.0614
14	MEIBOSCORE	1.19 ± 0.39	1.13 ± 0.34	0.0708
15	TOTAL MG SCORE (BE) (N - 105)	13.30 ± 4.73	12.97 ± 4.64	0.1971
16	Log MAR	0.217857 ± 0.083355	0.05 ± 0.099758	0.0005

The only TBUT and TMH showed correlation with the age (p=0.008 and 0.04), while rest parameters (including NIBUT) had no co-relationship. The objective parameters of dry eye disease were comparable in males and females except change in TMH. The cases with no systemic illness had significantly higher improvement in tear film stability (TBUT, NIBUT and Schirmer score) as compared to cases with systemic illness. (**Table 2**).

**Table 2:** Shows the relationship between age, gender, and systemic illness and TBUT, Schirmer test TMH, OSDI, and ocular surface staining score.

Parameters	Statistical significance	TBUT Change	Schirmer’s Change	TMH Change	NIBUT Change	OSDI Score Change	Staining Change
Age	r	-0.181	-0.003	-0.136	0.072	0.070	-0.091
	p	***0.008***	0.961	***0.049***	0.302	0.478	0.188
Males	P	>0.9999	>0.9999	***<0.0001***	***0.0026***	***<0.0001***	***<0.0001***
Females	p	***<0.0001***	***<0.0001***	>0.9999	***<0.0001***	***0.0141***	***<0.0001***
Systemic Illness	P	>0.9999	>0.9999	***<0.0001***	0.1226	***<0.0001***	***<0.0001***
No Systemic Illness	P	***<0.0001***	***<0.0001***	***0.0062***	***<0.0001***	***<0.0001***	***<0.0001***

TBUT, tear break up time; TMH, tear meniscus height; NIBUT, non-invasive break up time; OSDI, ocular surface disease index.

All the statistically significant (P<0.05) values are presented in bold.

The OSDI score improved significantly, from 23.22 ± 8.06 to 17.86 ± 8.62 (p<0.0001).

### Meibomian gland score

At the 1-month follow-up, the MG score of the lower lid had significantly decreased (p=0.002), while that of the upper lid remained comparable (p=0.38). The lid margin score improved for both the upper lid (p=0.03) and lower lid (p=0.07). MG expressibility also improved significantly in both the upper (p=0.0001) and lower lids (p=0.001). A slight improvement was observed in the meibum score (p=0.06) and the meiboscore (0.07). The total MG score improved from 13.3 ± 4.73 to 12.97 ± 4.64 at 1 month, though this was not statistically significant (p = 0.19).

**Figure 1:**
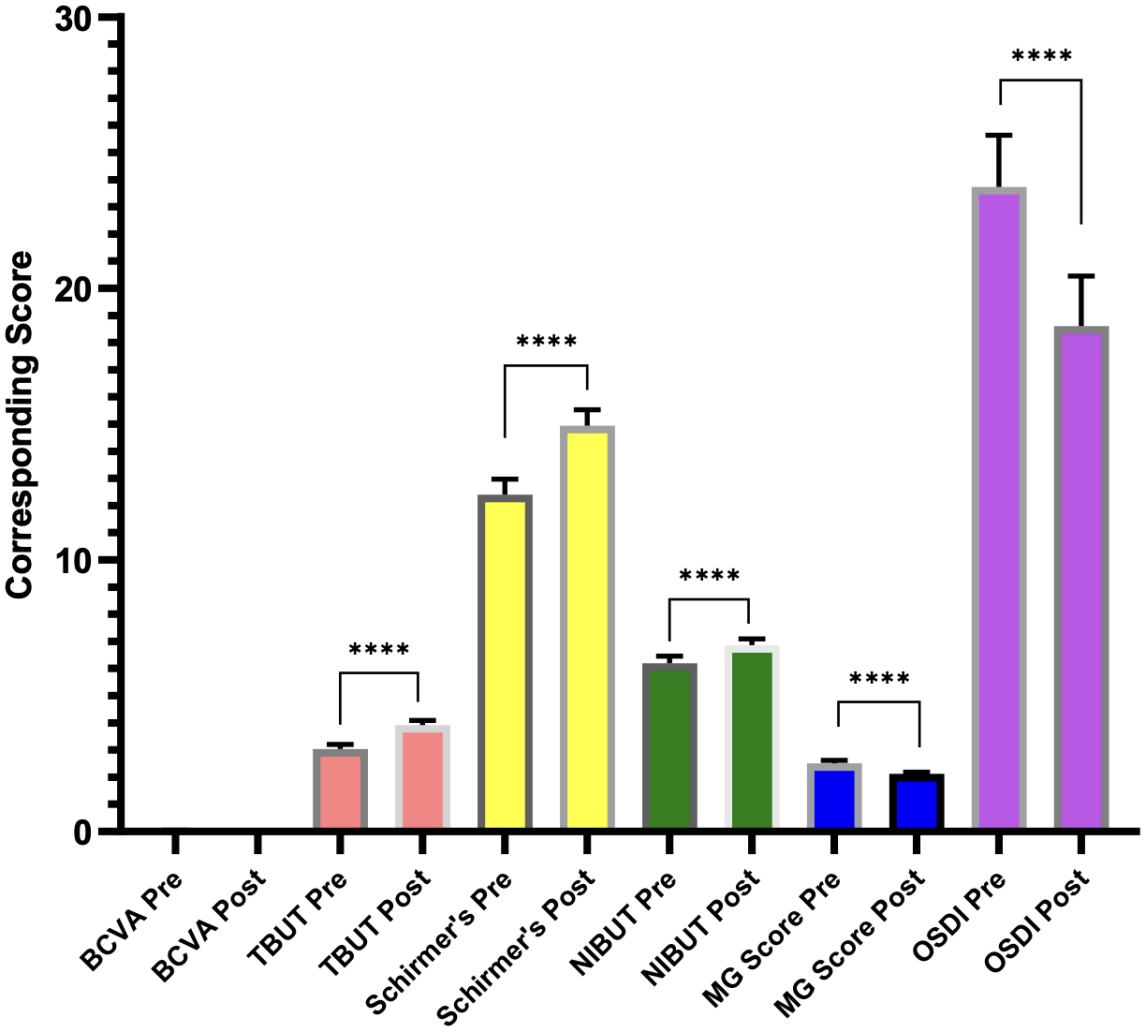
Graph showing improvement in dry eye disease parameters following the usage of Systane Complete eye drops. **** represents the difference between pre- and post-treatment values.

The improvement in all parameters is illustrated in **Figure 1**.

## Discussion

This is the first study evaluating the effectiveness of lipid nanoemulsion eye drops in the Indian population. Cases of dry eye disease were assessed in terms of improvement in dry eye disease parameters and Meibo score. There was a significant improvement in mean TBUT (p<0.0001), LLT (p<0.0001), and NIBUT (p<0.0001), suggesting that the lipid-based eye drops significantly improved tear film stability. This was also reflected in improved tear film parameters, including enhanced corneal staining scores (p<0.0001), mean Schirmer scores (p<0.0001), and mean TMH (p=0.0002).

In an *in vitro* study model, the presence of a lipid nanofilm containing 15% polar lipids on an electrolyte solution resulted in a substantial 57% reduction in the evaporation rate (16). Similar findings were reported in a review by Maulvi et al., which evaluated the efficacy of various lipid-based products—such as mineral oil, omega-3 fatty acids, phospholipids, and medium-chain triglycerides—in managing evaporative dry eye disease (17).

Older individuals have been observed to report fewer subjective symptoms and demonstrate more complete blink patterns compared to younger cohorts. Paradoxically, younger patients exhibit a greater degree of meibomian gland dropout and reduced mean lipid layer thickness (18). Furthermore, several studies have identified a negative correlation between age and both tear film break-up time and Schirmer’s test values, indicating a decline in tear film stability and aqueous production with advancing age. In contrast, parameters such as tear meniscus height and ocular surface staining scores have shown a positive correlation with age, reflecting age-associated alterations in ocular surface integrity (19). Notably, Zhao et al. reported that these age-related associations were significantly more pronounced in female patients, whereas no such trend was observed in males (20).

In this study, age and gender did not have a major association with improvement in dry eye disease parameters. This may be attributed to the inclusion of cases within a younger age group (18–55 years). However, there was marked improvement in individuals without systemic illness, particularly in terms of TBUT, Schirmer, and NIBUT scores, compared to those with systemic comorbidities. This suggests that lipid-based nanoemulsion was effective across both genders and all cases within the defined age range. Patients comorbidities may require stabilization.

Assessment of the MG score is an effective method for evaluating overall meibomian gland health. There was an improvement in the percentage of meibomian gland dropout in the upper lids (p=0.02) and lower lids (p=0.08), possibly reflecting improved health of partially compensated meibomian glands. There was an improved expressibility of the upper and lower lid meibomian glands (p<0.05), with comparable improvements in meibum quality (p=0.06) and meiboscore (p=0.07). The total MG score improved from 13.3 to 12.97, although this change was not statistically significant (p =0.19). This suggested that along with optimizing the tear film lipid, the lipid-based nano emulsion enhances the functionality of the meibomian glands. This was supplemented by subjective decrease in the symptoms of dry eye disease which was quantified by the change in mean OSDI score (p<0.0001) at one-month follow up. 

In a study by Craig et al., ninety-nine participants diagnosed with dry eye disease based on the TFOS DEWS II guidelines were randomized into two groups: one received lipid-based nanoemulsion drops (Systane COMPLETE) and the other non-lipid-based aqueous drops (Systane Ultra), administered four times a day for 6 months. There was a significant reduction in all symptom scores, including OSDI, DEQ-5, and SANDE (p<0.05 at 1 month) in both groups. Improvement in NIBUT and staining scores was observed at 4 months in both groups (p<0.05). The tear lipid layer increased from 3 months onward with only the lipid-based drops, specifically in cases with a suboptimal lipid layer (p=0.02). The authors concluded that although both formulations supported the treatment of mild-to-moderate dry eye disease, the lipid-based formulation was preferentially beneficial for patients requiring lipid-based supplementation (21).

In a similar study by Bickle et al., 119 cases of dry eye disease treated with PG-HPG eye drops were evaluated as part of post-market surveillance. The authors concluded that there were no adverse effects associated with the drop, and there was a statistically significant improvement in dry eye questionnaires, including the Impact of Dry Eye on Everyday Living—Symptom Bother (IDEEL-SB) and the Dry Eye Questionnaire (DEQ-5), with 1-month use (22).

A phase IV multicenter trial was conducted to assess the efficacy and safety of propylene glycol/hydroxypropyl guar (PG-HPG)-based nanoemulsion (Systane COMPLETE) in participants with dry eye disease. A total of 134 participants with various types of dry eye disease (aqueous-deficient, evaporative, and mixed) were included. There was an improvement in TBUT with a decrease in ocular symptoms. It was concluded that the drops were safe, effective, and well tolerated by individuals affected by dry eye disease and all its subtypes (12).

Most of the studies in the literature include cases of dry eye disease from Western populations (17). This is one of the first studies evaluating the efficacy of a lipid-based nanoemulsion in mild-to-moderate dry eye disease in Indian eyes.

Silverstein et al. showed that even a single drop of PG-HPG provided instant relief of dry eye disease symptoms (14). A study by Muntz et al. demonstrated that even in cases of dry eye disease induced by exposure to a prevalidated simulated adverse environment, the lipid-based nanoemulsion was superior in preserving tear film quality and alleviating symptoms (23).

The strengths of this study include that it is the first in the literature to evaluate the efficacy and safety of a lipid-based nanoemulsion in evaporative DED in the Indian population. It is also the first study to assess the role of the eye drop on the meibomian glands using objective scoring.

The limitations of the study include the absence of a control group and the relatively short follow-up duration. A comparison of the objective scores between the study group and a control group may have facilitated a more robust analysis of the results. In the future, a randomized controlled trial may help further evaluate the clinical outcomes of the formulation.

In conclusion, the lipid-based nanoemulsion of PG-HPG is a safe and effective option for managing evaporative DED. It also supports improved meibomian gland health and enhances the optimization of the ocular surface.

## Data Availability

The original contributions presented in the study are included in the article/**Supplementary Material**. Further inquiries can be directed to the corresponding author.

## Supplementary material

Supplementary Table 1 shows the median, mode, and inter-quartile range for TBUT, NIBUT, and Schirmer and their non-normal distribution.
