# Supplementary material for: To assess the effectiveness of Systane COMPLETE in improving meibomian gland score and in reducing symptoms of ocular dryness
Source: Front Ophthalmol (Lausanne). 2025 Nov 6;5:1577836. doi: 10.3389/fopht.2025.1577836 (PMC12631756; doi:10.3389/fopht.2025.1577836)
Supplement: Supplementary Table 1 — shows the median, mode, and inter-quartile range for TBUT, NIBUT, and Schirmer and their non-normal distribution. [file Table1.docx]

Supplementary table shows the median, mode and inter-quartile range for TBUT, TMH change and Schirmer and their non-normal distribution

|  | **TBUT** | | **Schirmer’s Change** | | **TMH Change** | |
| --- | --- | --- | --- | --- | --- | --- |
|  | Baseline | 1 Month | Baseline | 1 Month | Baseline | 1 Month |
| **Median** | 3 | 4 | 10 | 15 | 0.21 | 0.22 |
| **Mode** | 2 | 4 | 10 | 35 | 0.19 | 0.22 |
| **Inter-quartile range** | 2 | 2 | 12 | 20 | 0.08 | 0.07 |

*[TBUT: tear break up time; TMH: tear meniscus height]*
